# Linking the westernised oropharyngeal microbiome to the immune response in Chinese immigrants

**DOI:** 10.1186/s13223-020-00465-7

**Published:** 2020-07-25

**Authors:** Jing Guo, Xiaoping Zhang, Aarti Saiganesh, Christopher Peacock, Shu Chen, Gary A. Dykes, Belinda J. Hales, Peter N. Le Souëf, Guicheng Zhang

**Affiliations:** 1 School of Public Health, Curtin University, Perth, WA Australia; 2 Centre for Genetic Origins of Health and Disease, Curtin University and the University of Western Australia, Perth, WA Australia; 3 China National Bamboo Research Centre, Key Laboratory of Resources and Utilization of Bamboo of State Forestry Administration, Hangzhou, Zhejiang China; 4 Telethon Kids Institute, The University of Western Australia, Perth, WA Australia; 5 Marshall Centre for Infectious Disease, School of Pathology and Laboratory Medicine, The University of Western Australia, Perth, WA Australia; 6 Division of Cardiovascular and Respiratory Sciences, The University of Western Australia, Perth, WA Australia; 7 Curtin Health Innovation Research Institute, Curtin University, Perth, WA Australia

**Keywords:** Allergy and Immunology, Immigrants, Innate immune response, Microbiome, Toll-Like Receptors

## Abstract

**Background:**

Human microbiota plays a fundamental role in modulating the immune response. Western environment and lifestyle are envisaged to alter the human microbiota with a new microbiome profile established in Chinese immigrants, which fails to prime the immune system. Here, we investigated how differences in composition of oropharyngeal microbiome may contribute to patterns of interaction between the microbiome and immune system in Chinese immigrants living in Australia.

**Methods:**

We recruited 44 adult Chinese immigrants: newly-arrived (n = 22, living in Australia < 6 months) and long-term Chinese immigrants (n = 22, living in Australia > 5 years), with age and gender matched. Oropharyngeal swabs, serum and whole blood were collected. The 16 s ribosomal RNA gene from the swabs was sequenced on the Illumina MiSeq platform. Innate immune responses were determined by 23 Toll-like receptors (TLR) pathway cytokines, while adaptive immune responses were determined by IgG-associated response to specific microbial/viral pathogens.

**Results:**

The relative abundance of the genus *Leptotrichia* was higher in long-term immigrants as compared to that in newly-arrived Chinese immigrants, while the genus *Deinococcus* was significantly lower in long-term Chinese immigrants. The genera uncultured *Lachnospiraceae*, *Erysipelotrichaceae UCG*-*007*, *Veillonella*, and *Actinomycetales_ambiguous taxa* were negatively correlated with cytokine IL-6 in long-term Chinese immigrants (rho range: − 0.46 ~ − 0.73). With respect to adaptive immunity, several microbial taxa were significantly associated with IgG1 responsiveness to microbial antigens in long-term immigrants, while a significant correlation with IgG1 responsiveness to viral antigens was detected in newly-arrived immigrants.

**Conclusions:**

The composition of the oropharyngeal microbiome varies between newly-arrived and long-term Chinese immigrants. Specific microbial taxa are significantly associated with immunological parameters but with different association patterns between newly-arrived and long-term Chinese immigrants.

## Background

“Western-developed” vs. “Eastern-developing” gradients in many inflammatory conditions, such as asthma and allergic diseases, are significant [1, 2]. The International Study of Asthma and Allergies in Childhood confirmed these variations, showing that the self-reported asthma prevalence varied from 2 to 3% in developing countries to 20–40% in developed countries [3]. In addition, immigrants from a developing (Eastern) to a developed (Western) country are at an increased risk of asthma and allergy, with gradually increasing prevalence related to their years of residence in the developed country [4]. These differences in disease prevalence are thought to be related to lower levels of ‘hygiene’ in Eastern countries relative to Western countries [5], consistent with the ‘hygiene hypothesis’ [6]. Moreover, the possible risk factors could mediate their influence by epigenetic modifications [7]. Elucidation of the mechanisms underlying the inequality of asthma prevalence between Western and Eastern countries, and the increased trend of allergic conditions in immigrants from a low to a high asthma risk country, may hold the key to understanding why these conditions have increased in developed countries and are increasing in developing countries [8].

Microbes associated with humans play a pivotal role in maintaining human health [9]. Recent studies have shown that the microbiome is associated with diverse health conditions, such as inflammatory bowel diseases, autoimmune disorders, and allergy [10, 11]. All these conditions and diseases share a common chronic inflammatory mechanism associated with environmental and lifestyle risk factors [12]. Recent research emphasizes on microbiota-host interactions with the discovery that commensal microbes link the innate and adaptive response by producing small molecules [13]. It has been suggested that Western environments and lifestyles may alter human microbiota resulting in a microbiome profile that fails to prime the immune system. This in turn leads to chronic inflammation and causes a range of conditions that have increased during the past several decades in Western countries. It is therefore timely to investigate the influence of the Western environment and lifestyle on the human microbiome as well as the interaction between the microbiome and immune response in a homogeneous immigrant population to elucidate mechanisms underlying the development of asthma and other chronic inflammatory diseases.

Australia and China have one of the highest and lowest worldwide prevalence of asthma and allergies, respectively [3]. Chinese immigrants living in Australia have entered a ‘natural experimental environment’ with a high risk of developing asthma and allergy and provide a unique population to investigate microbiota changes resulting from the Western environment. We have found a marked shift in the innate and adaptive immune response between Chinese immigrants living in Australia for less than 6 months and more than 5 years. This was achieved by comparing the whole blood toll-like receptors (TLRs) pathway cytokine response and sera immunoglobulin G (IgG)-associated responsiveness to specific microbial/virus pathogens [14, 15]. Recently, our team reported that Chinese immigrant children living in Australia show different microbiome diversity and composition compared to Chinese-born Chinese children [16]. The aim of this study is to compare the oropharyngeal microbial profiles between newly-arrived (< 6 months) adult Chinese immigrants and long-term (> 5 years) adult Chinese immigrants. We also investigated the correlation between the oropharyngeal bacterial composition and innate immune response, adaptive immune response and atopic indices (serum IgE levels). An oropharyngeal swab was used as it is a non-invasive method and sampling creates little discomfort and also oropharyngeal microbiota have been successfully used to investigate the upper airway microbiome [17, 18]. This study contributes to an understanding of the Western environment-microbiome-chronic inflammation paradigm. The findings provide valuable insights into the treatment and prevention strategies for chronic inflammatory conditions such as asthma and allergy.

## Methods

### Study participants and design

**Fig. 1:**
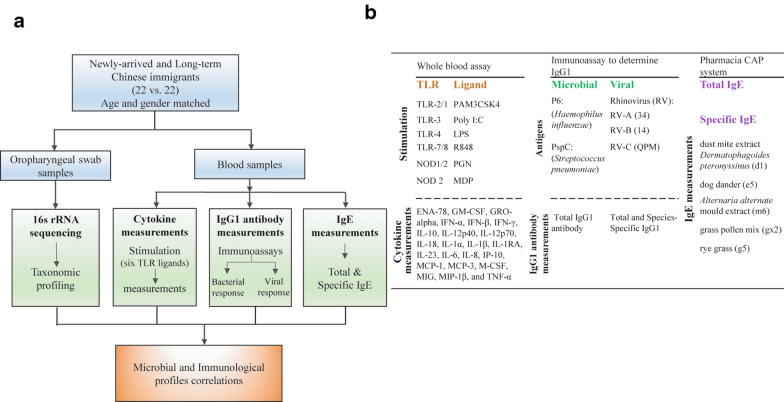
A whole study overview. **a** Study workflow. **b** The immune response measurements

We initiated a study on asthma and allergy in Chinese immigrants in Australia in 2012 [8]. This current study is part of a large Chinese immigrant study and the recruiting method has been described elsewhere [14]. Twenty-two newly-arrived Chinese immigrants (living in Australia < 6 months) and 22 long-term adult Chinese immigrants (living in Australia > 5 years) were recruited with age and gender matched over 3 month period (Nov 2014 to Feb 2015). We excluded participants that used antibiotics or had infections in the last 4 weeks. Most pairs of newly arrived and long-term Chinese immigrants were recruited on the same day and a few pairs were recruited within a two- or three-day days of each other. Oropharyngeal swab samples, serum, and whole blood samples were collected on the recruitment day. This study design followed the principles of the Declaration of Helsinki and was approved by the human research ethics committee at the Child and Adolescent Health Service (1969EP) and University of Western Australia (RA/4/1/6763). All participants gave informed written consent prior to participation. Oropharyngeal swabs were obtained using a sterile cotton swab which were immediately placed into a sterile collection tube. The swabs were sent to our laboratory on dry ice and kept frozen at − 80 °C freezer until further processing. A study overview is shown in Fig. 1.

### High-throughput 16S rRNA gene paired-end sequencing

DNA was extracted from pharyngeal swab samples using the QIAamp DNA Microbiome Kit (QIAGEN, cat#51704) following the manufacturer’s guidelines. PCR amplification and sequencing were performed by the Australian Genome Research Facility (AGRF). Briefly, universal 16S ribosomal RNA (rRNA) gene primers were used to amplify the V3–V4 region of the bacterial 16S rRNA gene of each sample, using AmpliTaq Gold 360 mastermix (Life Technologies, Australia) for the primary PCR. A secondary PCR to index the amplicons was performed with TaKaRa Taq DNA Polymerase (Clontech). The cycling conditions consisted of 95 °C for 7 min; 29 cycles of 94 °C for 30 s, 50 °C for 60 s, and 72 °C for 60 s; and then final extension at 72 °C for 7 min. The resulting amplicons were quantified using the fluorescent PicoGren assay and measured by fluorometry (Invitrogen Picogreen) and normalised. The eqimolar pool was then measured by qPCR (KAPA) followed by sequencing on the Illumina MiSeq (San Diego, CA, USA) with 2 × 300 base pairs paired-end chemistry. Negative (HPLC water) and positive (the BEI 16S gDNA mock community 782D) controls, as well as all samples, were sequenced on the same batch.

### Bioinformatics analysis

The output paired-end reads were merged using PANDASeq assembler [19] with the parameters at a minimum of 400 bp length and maximum of 500 bp length, and then analysed by downstream computational pipelines of the open source software package Quantitative Insights Into Microbial Ecology (QIIME v1.9.1) [20]. Chimera checking was performed with the usearch61 algorithm [21]. Sequences were clustered into Operational Taxonomic Units (OTUs) by UCLUST method [22] using the open-reference OTU-picking workflow pipelines against SILVA reference database (128 release), and a 97% similarity threshold. OTUs with abundance below 0.005% of the total number of sequences were discarded [23]. A total of 2516,134 sequences that passed the quality check were used in further analysis. An even depth of 2289 sequences per sample was used for alpha and beta diversity as all samples had at least this number of sequences. Alpha diversity was estimated using the chao1 richness estimate and Shannon index. Beta diversity was measured using the Bray–Curtis distance metric.

### Cytokine, IgG1 response to stimulation/antigens, and Immunoglobulin E (IgE) measurement

Twenty-three cytokines, representing the TLR innate immune response, were measured ex vivo using whole blood assay under stimulation of six TLR ligands (Fig. 1b). IgG1 responses to bacterial and viral antigens (Fig. 1b) that represent the adaptive immune response were tested using immunoassays for total IgG1 and immunoabsorption assays for species-specific IgG1 antibody binding. The detailed method was described in a previous paper [14]. The Pharmacia CAP system (Pharmacia Diagnostics AB, Uppsala, Sweden) was used to assess total and specific IgE (Fig. 1b) from serum samples at the PathWest Immunology Department (QEII Medical Centre, Perth, Australia). The specific IgE tests included house dust mite extract *Dermatophagoides pteronyssinus* (d1), dog dander (e5), *Alternaria alternate* mould extract (m6), grass pollen mix (g×2) and rye grass (g5).

### Statistical analysis

The relative abundance of bacterial taxa in samples from newly-arrived and long-term Chinese immigrants were compared using the Mann–Whitney U test (Statistical Package Social Science, SPSS, version 25.0). The linear discriminant analysis (LDA) effect size (LEfSe) method [24] was used to compare the microbial composition of the two Chinese immigrant groups. The compare_alpha_diversity.py script was used to compare the alpha diversity (within-sample diversity) metrics in the QIIME pipeline, which implements a nonparametric two-sample t-test with 999 Monte Carlo permutations. Beta diversity (between-sample diversity) comparisons were completed using analysis of similarities (ANOSIM) (compare_categories.py; QIIME). A value of *p* < 0.05 was considered statistically significant.

Principal component analysis (PCA) was used to extract the first PC representing the major variation of the six cytokine measurements (stimulated by six TLR ligands) for each of the 23 cytokines. The 23 cytokine PC1 scores were correlated with microbial taxa (over 1.0%) using Spearman correlation test. The microbial taxa (over 1.0%) were also correlated with IgG1 antibody responses to microbial and virus pathogens, and total and specific IgE for newly-arrived and long-term immigrants, respectively.

We also calculated the Spearman correlation of each microbial taxa (over 1.0%) with 138 cytokine measurements (23 cytokines * six TLR ligands) in those two immigrant groups, respectively. The correlation coefficients (ρ) calculated in long-term versus newly-arrived immigrants, respectively, were treated as a pair. Among the 138 pairs (as we have 138 cytokine measurements), those pairs with a ρ coefficient that is significant (*p *< 0.05) either in long-term or newly-arrived immigrants were selected. The Spearman ρ represents the correlation strength between taxa and innate immune response. A paired sample *t* test was used to test the difference in the strength of these correlations between the two immigrant groups in different taxonomic levels. All the *p* values were adjusted with Benjamini–Hochberg false discovery rate (FDR) correction.

## Results

### Oropharyngeal microbiota composition and diversity

**Fig. 2:**
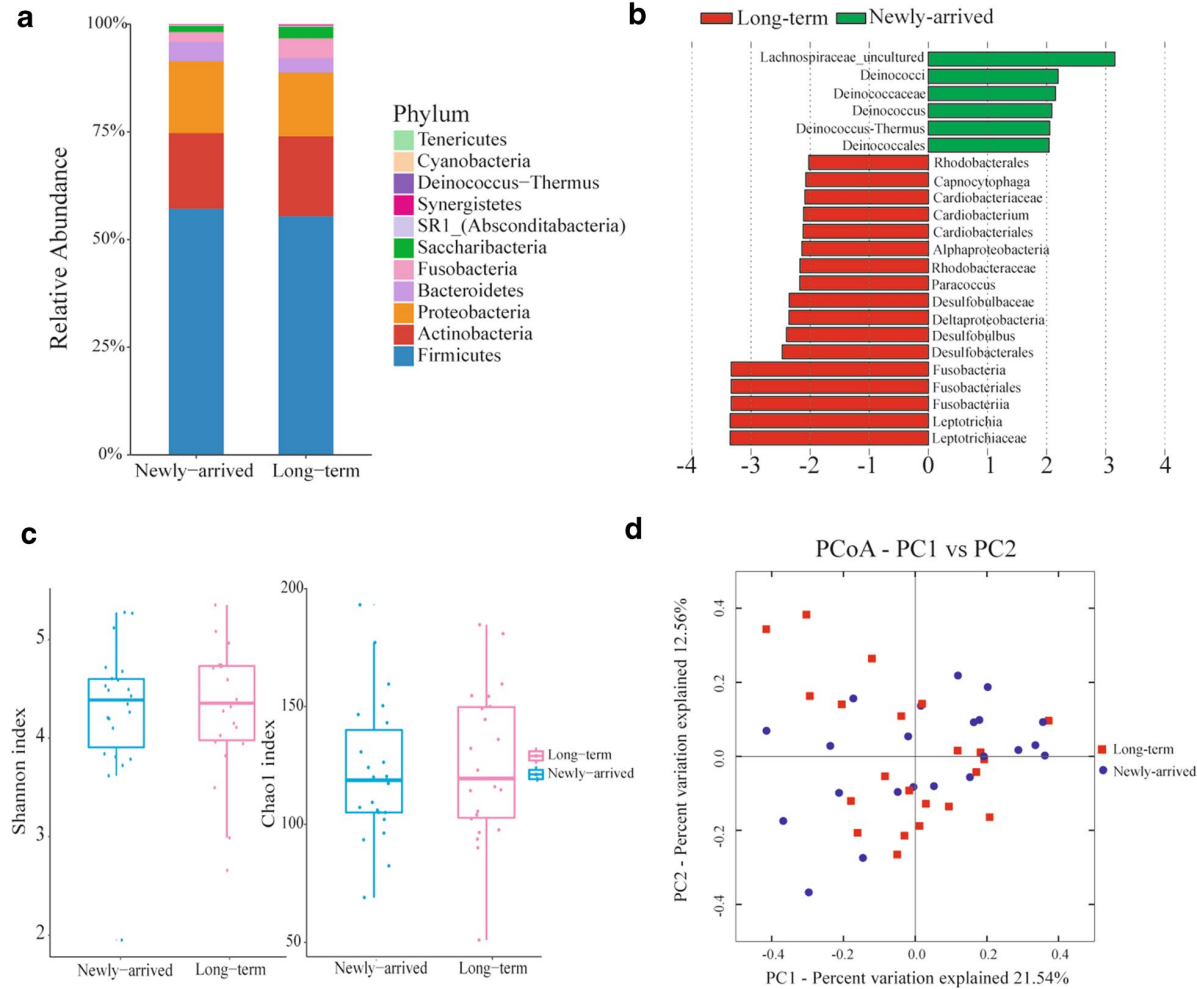
Comparison of the composition and diversity of newly-arrived and long-term Chinese immigrants. **a** The relative abundance of identified phyla in newly-arrived and long-term Chinese immigrants. The Y-axes represent relative phylum abundance and the x-axes represent sampling cohort, either from newly-arrived or long-term Chinese immigrants. **b** LEfSe analysis of microbiome changes. Taxa enriched in newly-arrived Chinese immigrants are indicated with a positive LDA score (green), and taxa enriched in long-term Chinese immigrants have a negative score (red). Only taxa meeting an LDA significant threshold of > 2 are shown. **c** Alpha diversity analysis among samples. Box-and-whisker plots of the Alpha diversities are exemplified by the Shannon and Chao1 index between newly-arrived (blue) and long-term (pink) Chinese immigrants. No difference was observed in Shannon and Chao1 indices. **d** Beta diversity analysis among samples was carried out according to the Bray–Curtis distance. Data points represent either long-term Chinese immigrant samples (red square) or new-arrived Chinese immigrants samples (blue dots), and the two major principle components are respectively represented on the x- and y- axes

Eleven bacterial phyla were detected in the oropharyngeal microbiome of newly-arrived and long-term Chinese immigrants (Fig. 2a, Additional file 1: Table S1). Firmicutes was dominant in both groups, comprising 54.3% and 53.4% of sequences in newly-arrived and long-term Chinese immigrants, respectively, followed by Actinobacteria, and Proteobacteria. At the genus level (Additional file 1: Table S2, Additional file 2: Fig. S1) there were 20 genera that account for 89.7% and 88.1% of the two groups respectively, with a minimum relative abundance of ≥ 1.0%. The relative abundance of Fusobacteria in newly-arrived immigrants (2.6%) was significantly lower than that of long-term immigrants (4.8%) (*p *< 0.01; FDR-corrected *p *= 0.066).

To further compare the microbial composition of long-term Chinese immigrants and newly-arrived Chinese immigrants, linear discriminant analysis effect size (LEfSe) was used (Fig. 2b, threshold of LDA score: 2). The relative abundance of the genus *Leptotrichia* was higher among long-term immigrants, compared to that of newly-arrived Chinese immigrants. However, the abundance of genus *Deinococcus* was significantly lower in long-term immigrants Chinese immigrants (Fig. 2b).

There was no significant difference in alpha diversity indices between the newly-arrived and long-term Chinese immigrants (Additional file 1: Table S3, and Fig. 2c). The Bray–Curtis distance measurer was applied to examine differences in bacterial community composition and structure between the newly-arrived and long-term Chinese immigrants (Fig. 2d). No significant difference was observed in beta diversity according to the Bray–Curtis distance measure (ANOSIM, *p *= 0.431).

### Oropharyngeal microbial taxa are significantly associated with immunological parameters

**Fig. 3:**
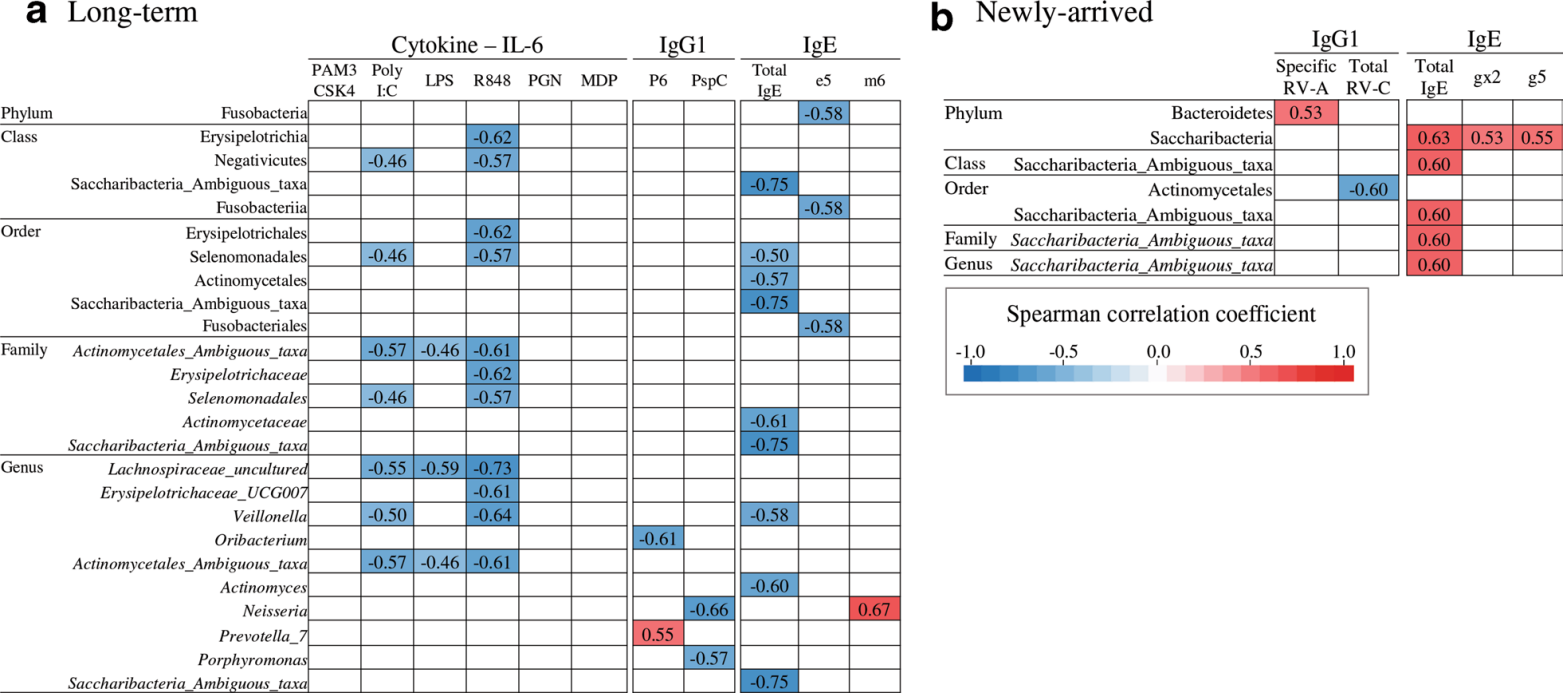
Significant correlation between oropharyngeal microbial taxonomies and immunological parameters (cytokine response, IgG1 antibody response, and IgE levels) among long-term Chinese immigrants (**a**) and newly-arrived Chinese immigrants (**b**). Spearman correlation test was used with Benjamini–Hochberg FDR correction (FDR-corrected *p *< 0.1). All taxa had a minimum relative abundance of ≥ 1.0% and only the taxa which had a significant correlation with at least one immunological parameters are displayed

A Spearman correlation was used to test for the association of bacterial taxa with innate immune responses, adaptive immune responses and IgE levels. Figure 3 summarizes the results with a significance (*p *< 0.1, the *p* values have been adjusted for multiple comparison). In long-term Chinese immigrants, 25 taxa significantly correlated with at least one of these immunological parameters, namely, cytokine IL-6, IgG1 binding to bacterial antigens, and IgE, with majority of them showing negative correlations. In newly-arrived Chinese immigrant, seven taxa were significantly correlated with at least one immunological parameters, namely, IgG1 binding to virus antigens and IgE, with majority of them demonstrating positive correlations.

Interestingly, negative microbial taxa correlations with PC1s of cytokine IL-6 were found exclusively significant among 11 taxa in long-term immigrants (rho range: − 0.60 ~ − 0.72). Further, Spearman correlation tests were also conducted between these microbial taxa and cytokine IL-6 stimulated by six TLR ligands (rho range: − 0.46 ~ −0.73, Fig. 3a). The taxa uncultured *Lachnospiraceae* (Firmicutes), *Erysipelotrichaceae UCG*-*007* (Firmicutes), *Veillonella* (Firmicutes), and *Actinomycetales_ambiguous taxa* (Actinobacteria) were negatively correlated with IL-6 stimulated by TLR ligands of Poly I:C, R848 and/or LPS.

In long-term Chinese immigrants the genera *Oribacterium* (Firmicutes), *Neisseria* (Proteobacteria) and *Porphyromonas* (Bacteroidetes) had a negative correlation with IgG1 antibody responses to bacterial antigen P6 or PspC, whereas *Prevotella 7* (Bacteroidetes) was positively correlated with P6 (Fig. 3a). In newly-arrived Chinese immigrants the phylum Bacteroidetes was positively correlated with viral specific antigen to RV-A, but the order Actinomycetales (Actinobacteria) was negatively correlated with viral total antigen to RV-C (Fig. 3b).

A total of 12 taxa were significantly negatively correlated with total IgE or specific IgE to dog dander, while only the genus *Neisseria* was positively correlated with mould extract in long-term immigrants. The genus *Saccharibacteria_Ambiguous* was negatively correlated with total IgE in long-term immigrants, but the phylum Saccharibacteria was positively associated with total IgE, specific IgE for grass pollen mix and rye grass in newly-arrived immigrants (Fig. 3).

### Different microbial taxonomies and cytokine correlation comparison between long-term and newly-arrived Chinese immigrants

**Fig. 4:**
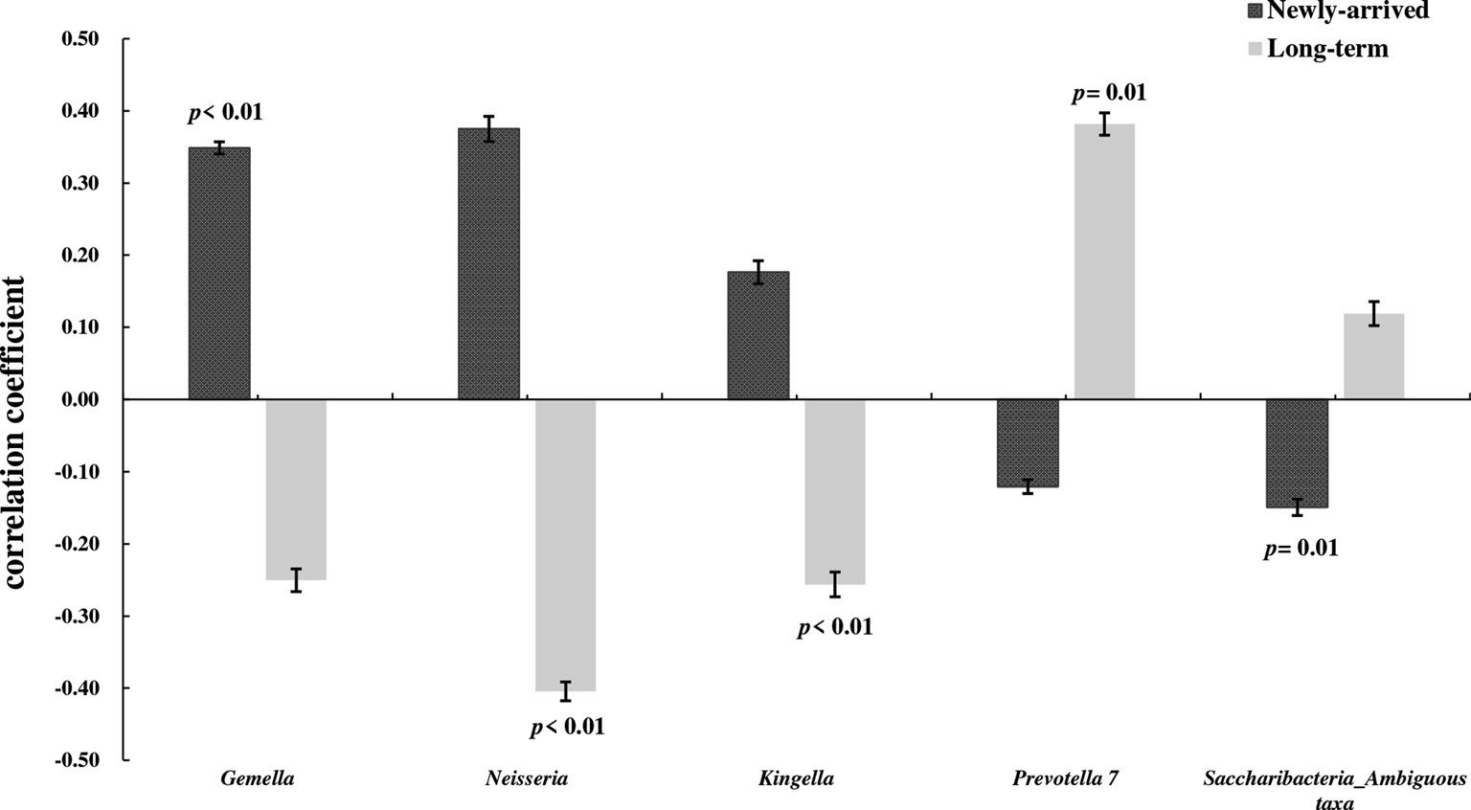
Comparison of the correlation strength of bacterial genera (over 1.0%) and the overall innate immune response (138 cytokine measurements) between newly-arrived and long-term Chinese immigrants. Y-axes represents the spearman correlation coefficients. The paired-sample *t* test was used to test the correlation strength difference. Only genera significantly different between the two groups were displayed

Additional file 1: Table S4 shows the correlation strengths between these bacterial taxa and the overall innate immune response (represented by the 138 cytokine measurements) in newly-arrived and long-term Chinese immigrants, respectively. Correlation strength of bacteria with overall innate response was significantly different between newly-arrived and long-term Chinese immigrants after adjusting for multiple comparisons. Correlation trends for the genera *Gemella* (Firmicutes), *Neisseria* (Proteobacteria) and *Kingella* (Proteobacteria) were positive in newly-arrived Chinese immigrants, but negatively associated with long-term immigrants. Conversely, *Prevotella 7* (Bacteroidetes) and *Saccharibacteria_Ambiguous* (Saccharibacteria) had negative correlation with the innate-immunity of newly-arrived Chinese immigrants, but positive correlation with that of long-term immigrants (Fig. 4).

## Discussion

To our knowledge this is the first study to investigate the oropharyngeal microbiome and its correlation with the immune response and IgE sensitization in an ethnically homogeneous immigrant population in Australia. In this study we use the phrase ‘Western environment’ as a collective term to denote the socio-cultural, physical and geographical environment in developed countries, such as the United States and Australia, and the ‘Eastern environment’ as a collective term to denote developing counties, such as China. Our analysis indicates that the variation of oropharyngeal microbial composition existed between newly-arrived (exposed to an Eastern environment) and long-term (exposed to a Western environment) Chinese immigrants. It is evidenced that the oropharyngeal microbiome had significant correlations with immune response and atopic sensitization, with distinctive association profiles present in newly-arrived and long-term Chinese immigrants.

The increased prevalence of allergic diseases in Westernised countries suggests that environment plays a vital role [25]. In this cohort, long-term Chinese immigrants living in Australia with a Western environment had more allergic symptoms compared with age and gender matched newly-arrived Chinese immigrants [14]. The increased allergy in long-term Chinese immigrants reported in our population and the substantial West versus East gradient of allergy prevalence between developed and developing countries indicates that the Western environment/lifestyle is the cause of allergic conditions. How or through what intermediates the Western environment causes allergies is poorly understood. With a population of 100 trillion cells, the human microbiome outnumbers human cells by at least a factor of ten [26]. A rising understanding of the fundamental role of the microbiota in the immune system has taken a centre stage in the field of immunology in decades. It is envisaged that environmental-host-microbial interaction should be critical for the development of allergic diseases. We surmise that the Western environment in Australia has altered the oropharyngeal microbiome in long-term Chinese immigrants, which leads to modifications of their innate and adaptive immunity, resulting in the development of allergies.

Our study found that individual pharynx microbiomes were dominated by phyla Firmicutes, Actinobacteria or Proteobacteria in Chinese immigrants, which is consistent with previous reports [27, 28]. Some differences in microbiome profiles between newly-arrived and long-term Chinese immigrants were found, which may partly be attributed to the Western vs Eastern environmental influence. The relative abundance of the genus *Leptotrichia* (Fusobacteria) was higher among long-term Chinese immigrants, compared to newly-arrived Chinese immigrants. The genus *Leptotrichia* is an anaerobic, Gram negative bacillus and part of the normal human oral flora. It may occasionally causes disease in immunocompromised hosts [29]. An asthma-related study reported that *Leptotrichia* was commonly detected in patients with corticosteroid-resistant asthma [30]. A recent study showed that *Leptotrichia* was strongly associated with mite sensitization as well as asthma [31]. In contrast to the Gram-negative *Leptotrichia*, *Deinococcus*, which is a Gram-positive genus, decreased in their relative abundance in long-term Chinese immigrants. *Deinococcus* is highly resistant to environmental hazards and its association with disease is rarely investigated, although there are a few studies that investigated its immunogenicity [32, 33]. Considering the difference between the environments in Australia and China and the large time difference (10 years) of residence in Australia, changes in the microbiome are expected. However, limited sample size and statistical power have precluded the identification of more taxa with a difference that is statistically significant after adjusting for the multiple comparisons. It is expected that a comparison study with a large sample size will identify more bacteria that have significantly different relative abundances between newly arrived and long-term immigrants.

Microbiome diversity has been associated with asthma and allergy [34, 35]. De Filippo et al. [36] found that the Chao1 and Shannon’s index in gut microbiome from Burkina Faso were higher (*p *< 0.01) than in EU samples at the OTU cutoff 0.10. Lin et al. [37] found Bangladeshi children exhibited greater gut microbiome diversity than the U.S. children. These two studies may support the assumption that the Western environment (such as Europe and USA) has a decreased microbiome diversity, which may affect the development of asthma and allergy in Western countries. We initially hypothesized Chinese immigrants would have decreased airway microbiome diversity after living in the Western environment for a sufficient period of time. However, our result shows no difference between newly-arrived and long-term Chinese immigrants in alpha and beta diversity of oropharyngeal microbiota. Therefore, this initial hypothesis was not confirmed. However, considering that the 16S sequencing method is measuring the relative abundance rather than absolute taxa, our findings do not conflict with the assumption that there may be less microbiome taxa in terms of copy numbers of bacteria existing in the human pharynx, in the long-term relative to newly-arrived Chinese immigrants. The assumption was based on the premise that hygienic environments are more prevalent in Western countries relative to Eastern countries. The copy numbers of bacterial taxa in the human pharynx need to be further investigated with a focus on comparing Western versus Eastern environments.

The immune system is made up of a complex network of innate and adaptive responses capable of adapting to highly-diverse challenges [38]. The innate immune system is the first line of defence against pathogens and acts by detecting conserved microbial structures through TLRs. Cumulative studies revealed, in Westernised countries, changes in diets/lifestyle, overuse of antibiotics, removal of parasitic infections, may have resulted in a lack of microbiome resilience and diversity to establish balanced immune responses [10]. Our previous study indicated that long-term Chinese immigrants had attenuated innate cytokine responses [15]. Present study found a significant correlation between the relative abundance of several bacterial and immune response measurements, such as TLR pathway cytokines, specific IgE and bacterial or virial antibody response. This indicates that the human microbiome has an influence on host immune response. In long-term Chinese immigrant IL6 was negatively correlated with several bacterial genera from the phyla Firmicutes and Actinobacteria. The correlations were more apparent for IL-6 stimulated by viral (TLR3, TLR7/8) and/or Gram-negative bacterial (TLR4) ligands. In a cross-sectional investigation it is not possible to determine a cause-effect relationship. A further investigation is warranted to determine if these bacteria have inhibited the whole blood IL-6 response in the TLR pathway or if the IL-6 response has prohibited the proliferation of these taxa in Chinese immigrants living in a Western environment for a significant period of time. Importantly, the present study has identified a convincing link between the oropharyngeal microbiome and innate and adaptive immunity in the Chinese immigrant population. It should be highlighted that these interactions of the microbiome and the overall innate immune responses are distinctly different in newly-arrived and long-term Chinese immigrants. Considering our previous findings that a stronger positive correlation between innate and adaptive responses were apparent among newly-arrived Chinese immigrants [14], we currently demonstrate that genera from the dominant phyla (Firmicutes, and Proteobacteria) were positively correlated with the overall innate immune response among newly-arrived Chinese immigrant and conversely with negative correlation in long-term immigrants. These complex interactions, which may occur in either direction, between human microbiomes and innate and adaptive immune response in a Western versus Eastern environment is expected to account for the uneven distribution of asthma and allergies in Western and Eastern world.

One of the limitations of the study is the sample size. After adjusting for multiple comparisons, only a few taxa were found to have a significant difference between newly-arrived and long-term Chinese immigrants. However, in such a study with a relatively small sample size we have convincingly identified the link between human microbiomes and immune response after we have strictly adjusted for multiple tests. This suggests a robust relationship between the microbiome and immune response. Secondly, this is a cross-sectional study that cannot infer a cause-effect relationship. Future longitudinal studies on newly arrived Chinese immigrants in a Western country that continually scrutinises the changes in diets, microbiomes, and immune responses for several years would be ideal. In addition, a matched Australian control group is required.

## Conclusion

The relative abundance of the genus *Leptotrichia* was higher among long-term immigrants, compared to that of newly-arrived Chinese immigrants. However, the abundance of the genus *Deinococcus* was significantly lower in long-term Chinese immigrants. Specific microbial taxa are significantly associated with immunological parameters but with different association patterns between newly-arrived and long-term Chinese immigrants. In addition, the correlations between the microbiome and the overall innate immune responses are distinctly different in newly-arrived and long-term Chinese immigrants. There is a strong bond between oropharyngeal microbiome and host immune response. Our study identifies a novel avenue to determine the associations of these changes in human microbiota with common chronic diseases such as asthma and allergy in the Western environment.

## Supplementary information

**Additional file 1: Table S1.** The phylum level of oropharyngeal microbiome compared between newly-arrived and long-term Chinese immigrants. **Table S2.** The genus level of oropharyngeal microbiome compared between newly-arrived and long-term Chinese immigrants. **Table S3.** Alpha diversity metrics for pharyngeal swab samples collected from newly arrived and long-term Chinese immigrants. Values represent mean ± SD. **Table S4.** The comparison of taxa correlations with innate immune response between newly-arrived and long-term Chinese immigrants (paired sample *t* test).

**Additional file 2: Fig. S1.** The relative abundance of identified genera in newly-arrived and long-term Chinese immigrants. Y-axes represent relative genus abundance and x-axes represent sampling cohort, either from newly-arrived or long-term Chinese immigrants. Only the genera with relative abundance ≥1.0% were listed, and genera with relative abundances less than 1.0% were combined into the ‘other’ category.

## Data Availability

The data that support the findings of this study are not publicly available due to the undergoing of more analysis but are available from the corresponding author upon reasonable request.

## Abbreviations

rRNA: Ribosomal RNA
OTU: Operational Taxonomic Unit
LDA: The linear discriminant analysis
LEfSe: The linear discriminant analysis effect size
PCA: Principal component analysis
FDR: False discovery rate
