# Supplementary material for: Linking the westernised oropharyngeal microbiome to the immune response in Chinese immigrants
Source: Allergy Asthma Clin Immunol. 2020 Jul 25;16:67. doi: 10.1186/s13223-020-00465-7 (PMC7491349; doi:10.1186/s13223-020-00465-7)
Supplement: Supplementary file 1 — Additional file 1: Table S1. The phylum level of oropharyngeal microbiome compared between newly-arrived and long-term Chinese immigrants. Table S2. The genus level of oropharyngeal microbiome compared between newly-arrived and long-term Chinese immigrants. Table S3. Alpha diversity metrics for pharyngeal swab samples collected from newly arrived and long-term Chinese immigrants. Values represent mean ± SD. Table S4. The comparison of taxa correlations with innate immune response between newly-arrived and long-term Chinese immigrants (paired sample t test). [file 13223_2020_465_MOESM1_ESM.docx]

**Table S1. The phylum level of oropharyngeal microbiome compared between newly-arrived and long-term Chinese immigrants**

| Phylum | Test-Statistic | *FDR_P^*^* | Newly-arrived immigrants | Long-term immigrants |
| --- | --- | --- | --- | --- |
| Firmicutes | 0.243 | 0.781 | 54.32% | 53.42% |
| Actinobacteria | 0.344 | 0.781 | 17.71% | 18.34% |
| Proteobacteria | 0.220 | 0.781 | 18.17% | 16.81% |
| Fusobacteria | 7.542 | 0.066 | 2.63% | 4.80% |
| Bacteroidetes | 0.009 | 0.925 | 3.95% | 3.49% |
| Saccharibacteria | 0.838 | 0.779 | 2.19% | 2.50% |
| Synergistetes | 1.620 | 0.745 | 0.60% | 0.39% |
| SR1 (Absconditabacteria) | 0.637 | 0.779 | 0.31% | 0.20% |
| Cyanobacteria | 0.065 | 0.878 | 0.02% | 0.04% |
| Tenericutes | 0.808 | 0.779 | 0.01% | 0.00% |
| Deinococcus-Thermus | 4.065 | 0.241 | 0.09% | 0.00% |
| *: The p-values were FDR-corrected to control for multiple testing. | | | | |

**Table S2. The genus level of oropharyngeal microbiome compared between newly-arrived and long-term Chinese immigrants**

| Genus | Test-Statistic | *FDR_P^*^* | Newly-arrived immigrants | Long-term immigrants |
| --- | --- | --- | --- | --- |
| *Streptococcus*  (Firmicutes; Bacilli; Lactobacillales; Streptococcaceae) | 0.014 | 0.953 | 29.55% | 31.07% |
| *Rothia* (Actinobacteria; Actinobacteria; Micrococcales; Micrococcaceae) | 0.926 | 0.715 | 9.76% | 9.93% |
| *Veillonella* (Firmicutes; Negativicutes; Selenomonadales; Veillonellaceae) | 0.600 | 0.763 | 9.69% | 8.38% |
| *uncultured* (Proteobacteria; Betaproteobacteria; Neisseriales; Neisseriaceae) | 0.267 | 0.853 | 6.16% | 5.02% |
| *Leptotrichia* (Fusobacteria; Fusobacteriia; Fusobacteriales; Leptotrichiaceae) | 6.308 | 0.398 | 2.38% | 4.61% |
| *Haemophilus* (Proteobacteria; Gammaproteobacteria; Pasteurellales; Pasteurellaceae) | 0.108 | 0.895 | 3.84% | 4.25% |
| *Actinomyces* (Actinobacteria; Actinobacteria; Actinomycetales; Actinomycetaceae) | 0.014 | 0.953 | 4.08% | 4.22% |
| *Neisseria* (Proteobacteria; Betaproteobacteria; Neisseriales; Neisseriaceae) | 0.754 | 0.755 | 6.56% | 3.29% |
| *Gemella* (Firmicutes; Bacilli; Bacillales; Family XI) | 1.607 | 0.715 | 1.79% | 3.05% |
| Ambiguous_taxa (Saccharibacteria; Ambiguous_taxa; Ambiguous_taxa; Ambiguous_taxa) | 0.564 | 0.767 | 1.99% | 2.28% |
| Oribacterium (Firmicutes; Clostridia; Clostridiales; Lachnospiraceae) | 0.055 | 0.936 | 2.07% | 2.09% |
| Ambiguous_taxa (Actinobacteria; Actinobacteria; Actinomycetales; Ambiguous_taxa) | 2.548 | 0.649 | 0.69% | 2.04% |
| Kingella (Proteobacteria; Betaproteobacteria; Neisseriales; Neisseriaceae) | 1.110 | 0.715 | 0.21% | 1.66% |
| *Granulicatella* (Firmicutes; Bacilli; Lactobacillales; Carnobacteriaceae) | 1.166 | 0.715 | 1.91% | 1.56% |
| Porphyromonas (Bacteroidetes; Bacteroidia; Bacteroidales; Porphyromonadaceae) | 1.141 | 0.715 | 1.00% | 1.29% |
| Prevotella 7 (Bacteroidetes; Bacteroidia; Bacteroidales; Prevotellaceae) | 0.927 | 0.715 | 1.89% | 1.13% |
| uncultured (Firmicutes; Clostridia; Clostridiales; Lachnospiraceae) | 5.446 | 0.398 | 2.04% | 0.63% |
| Corynebacterium 1 (Actinobacteria; Actinobacteria; Corynebacteriales; Corynebacteriaceae) | 1.237 | 0.715 | 1.54% | 0.38% |
| Erysipelotrichaceae UCG-007 (Firmicutes; Erysipelotrichia; Erysipelotrichales; Erysipelotrichaceae) | 1.886 | 0.715 | 1.52% | 0.92% |
| Selenomonas 3 (Firmicutes; Negativicutes; Selenomonadales; Veillonellaceae) | 0.007 | 0.957 | 1.06% | 0.29% |
| *: The p-values were FDR-corrected to control for multiple testing. | | | | |

**Table S3. Alpha diversity metrics for pharyngeal swab samples collected from newly arrived and long-term Chinese immigrants. Values represent mean ± SD.**

|  | Newly-arrived | Long-term | *p* value |
| --- | --- | --- | --- |
| Observed OTUs | 83.58±11.86 | 92.03±18.56 | 0.094 |
| Chao1 richness estimate | 121.82±29.06 | 124.97±31.72 | 0.732 |
| Faith's phylogenetic diversity | 3.60±1.44 | 3.63±1.45 | 0.943 |
| Shannon's index | 4.27±0.68 | 4.29±0.64 | 0.907 |
| Simpson’s index | 0.88±0.10 | 0.89±0.09 | 0.82 |

**Table S4. The comparison of taxa correlations with innate immune response between newly-arrived and long-term Chinese immigrants (paired sample *t* test)**

|  | Taxa | number of test | mean of ρ newly-arrvied Immigrants | mean of ρ long-term Immigrants | mean difference of ρ | *p* | *q^*^* |
| --- | --- | --- | --- | --- | --- | --- | --- |
| Phylum | Proteobacteria | 5 | 0.45 | -0.11 | 0.55 | 0.00 | 0.00 |
|  | Bacteroidetes | 10 | 0.37 | 0.03 | 0.33 | 0.01 | 0.01 |
|  | Saccharibacteria | 20 | -0.11 | 0.16 | -0.27 | 0.01 | 0.01 |
|  | Firmicutes | 8 | -0.06 | -0.18 | 0.12 | 0.21 | 0.31 |
|  | Actinobacteria | 11 | -0.21 | -0.01 | -0.19 | 0.28 | 0.33 |
|  | Fusobacteria | 3 | 0.10 | 0.19 | -0.10 | 0.77 | 0.77 |
| Class |  |  |  |  |  |  |  |
|  | Bacilli | 8 | 0.05 | -0.27 | 0.32 | 0.01 | 0.11 |
|  | Betaproteobacteria | 10 | 0.37 | 0.01 | 0.37 | 0.02 | 0.11 |
|  | Actinobacteria | 8 | 0.17 | -0.06 | 0.23 | 0.30 | 0.66 |
|  | Coriobacteriia | 13 | -0.23 | -0.04 | -0.19 | 0.24 | 0.66 |
|  | Erysipelotrichia | 30 | -0.29 | -0.17 | -0.12 | 0.29 | 0.66 |
|  | Bacteroidia | 8 | 0.02 | 0.15 | -0.13 | 0.58 | 0.91 |
|  | Clostridia | 3 | -0.32 | -0.04 | -0.28 | 0.53 | 0.91 |
|  | Negativicutes | 16 | -0.27 | -0.26 | -0.02 | 0.90 | 1.00 |
|  | Fusobacteriia | 3 | 0.10 | 0.19 | -0.10 | 0.77 | 1.00 |
| Order |  |  |  |  |  |  |  |
|  | Bacillales | 23 | 0.34 | -0.25 | 0.59 | 0.00 | 0.00 |
|  | Neisseriales | 15 | 0.34 | -0.03 | 0.38 | 0.00 | 0.01 |
|  | Pasteurellales | 3 | 0.04 | 0.49 | -0.44 | 0.00 | 0.01 |
|  | Saccharibacteria_Ambiguous_taxa | 26 | -0.15 | 0.12 | -0.27 | 0.00 | 0.01 |
|  | Corynebacteriales | 8 | 0.18 | -0.24 | 0.43 | 0.03 | 0.09 |
|  | Micrococcales | 10 | 0.31 | -0.05 | 0.35 | 0.07 | 0.15 |
|  | Coriobacteriales | 13 | -0.23 | -0.04 | -0.19 | 0.24 | 0.47 |
|  | Lactobacillales | 3 | -0.05 | -0.14 | 0.08 | 0.30 | 0.47 |
|  | Erysipelotrichales | 30 | -0.29 | -0.17 | -0.12 | 0.29 | 0.47 |
|  | Clostridiales | 3 | -0.32 | -0.04 | -0.28 | 0.53 | 0.73 |
|  | Bacteroidales | 8 | 0.02 | 0.15 | -0.13 | 0.58 | 0.73 |
|  | Actinomycetales | 14 | -0.27 | -0.30 | 0.03 | 0.79 | 0.85 |
|  | Fusobacteriales | 3 | 0.10 | 0.19 | -0.10 | 0.77 | 0.85 |
|  | Selenomonadales | 16 | -0.27 | -0.26 | -0.02 | 0.90 | 0.90 |
| Family |  |  |  |  |  |  |  |
|  | Family_XI | 21 | 0.35 | -0.25 | 0.60 | 0.00 | 0.00 |
|  | Neisseriaceae | 15 | 0.34 | -0.03 | 0.38 | 0.00 | 0.01 |
|  | Pasteurellaceae | 3 | 0.04 | 0.49 | -0.44 | 0.00 | 0.01 |
|  | Saccharibacteria_Ambiguous_taxa | 26 | -0.15 | 0.12 | -0.27 | 0.00 | 0.01 |
|  | Actinomycetaceae | 15 | -0.36 | -0.05 | -0.31 | 0.01 | 0.05 |
|  | Prevotellaceae | 17 | -0.04 | 0.28 | -0.33 | 0.02 | 0.05 |
|  | Corynebacteriaceae | 8 | 0.18 | -0.24 | 0.43 | 0.03 | 0.08 |
|  | Actinomycetales_Ambiguous_taxa | 10 | -0.04 | -0.40 | 0.36 | 0.04 | 0.08 |
|  | Micrococcaceae | 10 | 0.31 | -0.04 | 0.35 | 0.07 | 0.13 |
|  | Carnobacteriaceae | 10 | 0.29 | 0.10 | 0.18 | 0.16 | 0.29 |
|  | Coriobacteriaceae | 13 | -0.23 | -0.04 | -0.19 | 0.24 | 0.39 |
|  | Erysipelotrichaceae | 30 | -0.29 | -0.17 | -0.12 | 0.29 | 0.44 |
|  | Porphyromonadaceae | 5 | 0.17 | 0.40 | -0.23 | 0.37 | 0.51 |
|  | Streptococcaceae | 3 | -0.08 | -0.14 | 0.06 | 0.42 | 0.54 |
|  | Leptotrichiaceae | 3 | 0.24 | -0.02 | 0.26 | 0.51 | 0.61 |
|  | Lachnospiraceae | 6 | -0.16 | -0.29 | 0.13 | 0.60 | 0.68 |
|  | Peptostreptococcaceae | 5 | 0.20 | 0.28 | -0.09 | 0.72 | 0.77 |
|  | Veillonellaceae | 16 | -0.27 | -0.26 | -0.02 | 0.90 | 0.90 |
| Genus |  |  |  |  |  |  |  |
|  | Gemella | 21 | 0.35 | -0.25 | 0.60 | 0.00 | 0.00 |
|  | Neisseria | 14 | 0.38 | -0.40 | 0.78 | 0.00 | 0.00 |
|  | Prevotella_7 | 18 | -0.12 | 0.38 | -0.50 | 0.00 | 0.00 |
|  | Kingella | 18 | 0.18 | -0.26 | 0.43 | 0.00 | 0.01 |
|  | Saccharibacteria_Ambiguous_taxa | 26 | -0.15 | 0.12 | -0.27 | 0.00 | 0.01 |
|  | Rothia | 12 | 0.36 | -0.03 | 0.39 | 0.02 | 0.08 |
|  | Actinomycetales_Ambiguous_taxa | 10 | -0.04 | -0.40 | 0.36 | 0.04 | 0.10 |
|  | Actinomyces | 17 | -0.34 | -0.09 | -0.24 | 0.04 | 0.10 |
|  | Lachnospiraceae_uncultured | 12 | -0.13 | -0.42 | 0.29 | 0.09 | 0.19 |
|  | Corynebacterium_1 | 6 | 0.32 | -0.02 | 0.35 | 0.19 | 0.37 |
|  | Veillonella | 17 | -0.24 | -0.07 | -0.17 | 0.20 | 0.37 |
|  | Lachnospiraceae_uncultured | 8 | 0.14 | 0.32 | -0.18 | 0.30 | 0.50 |
|  | Streptococcus | 3 | -0.08 | -0.14 | 0.06 | 0.42 | 0.65 |
|  | Erysipelotrichaceae_UCG007 | 28 | -0.27 | -0.19 | -0.08 | 0.49 | 0.68 |
|  | Leptotrichia | 3 | 0.24 | -0.02 | 0.26 | 0.51 | 0.68 |
|  | Haemophilus | 3 | 0.03 | 0.28 | -0.25 | 0.63 | 0.79 |
|  | Atopobium | 5 | -0.17 | -0.25 | 0.08 | 0.79 | 0.83 |
|  | Porphyromonas | 7 | 0.32 | 0.36 | -0.04 | 0.83 | 0.83 |
|  | Granulicatella | 16 | 0.04 | 0.08 | -0.04 | 0.74 | 0.83 |
|  | Oribacterium | 5 | 0.07 | 0.17 | -0.10 | 0.75 | 0.83 |
| *: The *q* values were FDR-corrected *p* values to control for multiple testing. | | | | | | | |
