# Supplementary figures and images for: Linking the westernised oropharyngeal microbiome to the immune response in Chinese immigrants
Source: Allergy Asthma Clin Immunol. 2020 Jul 25;16:67. doi: 10.1186/s13223-020-00465-7 (PMC7491349; doi:10.1186/s13223-020-00465-7)

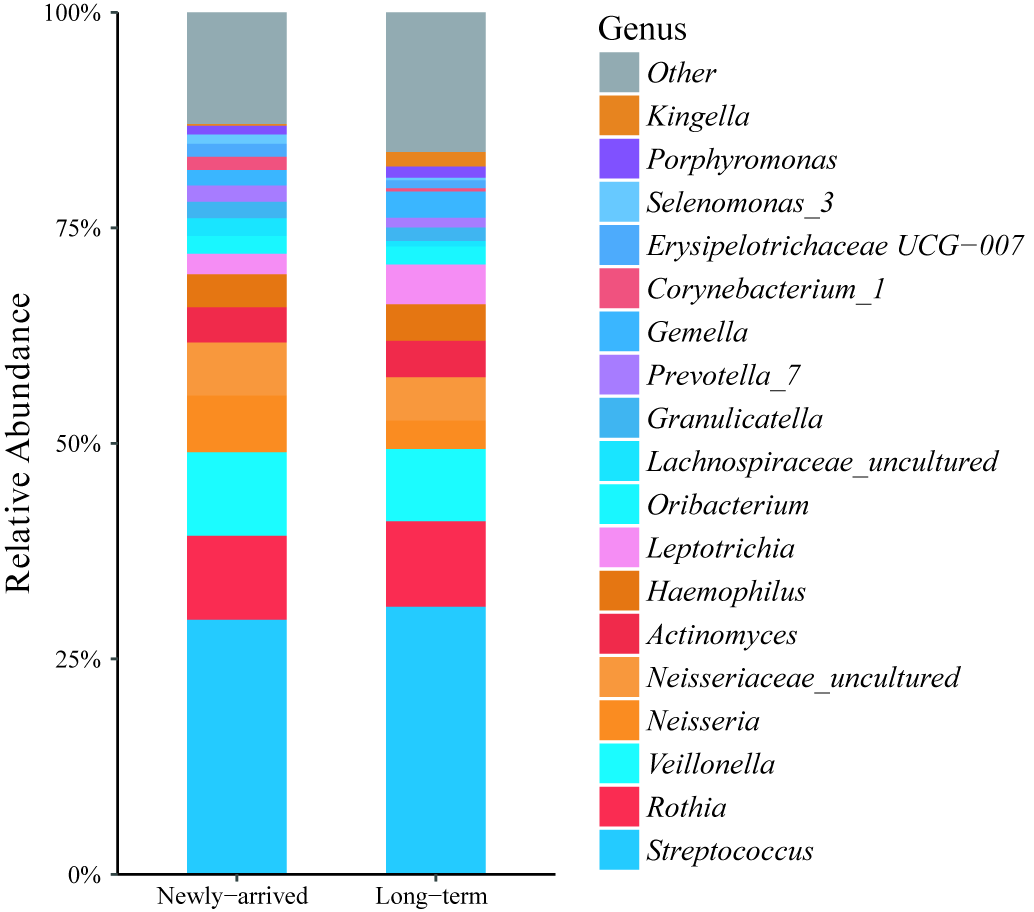

Supplement: Supplementary file 2 — Additional file 2: Fig. S1. The relative abundance of identified genera in newly-arrived and long-term Chinese immigrants. Y-axes represent relative genus abundance and x-axes represent sampling cohort, either from newly-arrived or long-term Chinese immigrants. Only the genera with relative abundance ≥1.0% were listed, and genera with relative abundances less than 1.0% were combined into the ‘other’ category. [file 13223_2020_465_MOESM2_ESM.tif]
